# Dual inhibition of HDAC and tyrosine kinase signaling pathways with CUDC-907 attenuates TGFβ1 induced lung and tumor fibrosis

**DOI:** 10.1038/s41419-020-02916-w

**Published:** 2020-09-17

**Authors:** Wentian Zhang, Yajie Zhang, Tian Tu, Sabastian Schmull, Yu Han, Wenbo Wang, Hecheng Li

**Affiliations:** 1 Department of Thoracic Surgery, Ruijin Hospital, Shanghai Jiao Tong University School of Medicine, 197 Ruijin 2nd Rd, Shanghai, 200025 China; 2 Department of Plastic and Reconstructive Surgery, Shanghai Ninth People’s Hospital, Shanghai Jiao Tong University School of Medicine, Shanghai Key Laboratory of Tissue Engineering, 639 ZhiZaoJu Road, Shanghai, 200011 China; 3 Renji-Med X Clinical Stem Cell Research Center, Renji Hospital, School of Medicine, Shanghai Jiao Tong University, No. 160 Pu-Jian Road, Shanghai, 200127 China

**Keywords:** Drug regulation, Target identification

## Abstract

TGFβ1 signaling is a critical driver of collagen accumulation in pulmonary fibrotic diseases and a well-characterized regulator of cancer associated fibroblasts (CAF) activation in lung cancer. Myofibroblasts induced by TGFβ1 and other factors are key players in the pathogenesis of lung fibrosis and tumor. Tremendous attention has been gained to targeting myofibroblasts in order to inhibit the progression of fibrosis and myofibroblast-induced tumor progression and metastasis. Here we determined the therapeutic efficacy of simultaneously targeting PI3K and HDAC pathways in lung myofibroblasts and CAF with a single agent and to evaluate biomarkers of treatment response. CUDC-907 is a first-in-class compound, functioning as a dual inhibitor of HDACs and PI3K/AKT pathway. We investigated its effects in counteracting the activity of TGFβ1-induced myofibroblasts/CAF in regard to cell proliferation, migration, invasion, apoptosis in vitro antifibrosis efficiency in vivo. We found that CUDC-907 inhibited myofibroblasts/CAF cell proliferation, migration and apoptosis in a dose-dependent manner and caused cell cycle arrest at G1-S phase. CUDC-907 not only inhibited myofibroblasts markers expression, but also significantly inhibited the phosphorylation level of AKT, mTOR, Smad2/3, and promoted acetylation of histones. Furthermore, the observed inhibitory effect was also confirmed in bleomycin-induced mice lung fibrosis and nude mouse transplanted tumor model. Overall, these data suggest that dual inhibition of HDAC and the tyrosine kinase signaling pathways with CUDC-907 is a promising treatment strategy for TGFβ1-induced lung and tumor fibrosis.

## Introduction

Pathological lung fibrosis, and its promoting effects on tissue stiffness, is a major cause of human morbidity and mortality^1^. TGF-β1 signaling is both an initiator and a driver of lung stiffness because of extracellular matrix (ECM) deposition and myofibroblasts differentiation. Approximately 80% of the upregulated genes in lungs of patients with idiopathic pulmonary fibrosis are reported to be direct or indirect TGF-β1 target genes^2^. Exaggerated TGF-β1 signaling is strongly implicated not only in lung fibrotic diseases, but also in cancer progression^3,4^.

Myofibroblasts, induced by TGFβ1-mediated signaling, exhibit continued activation in proliferation, anti-apoptosis, migration, invasion as well as expressing collagens and myofibroblasts markers such as α-SMA, FAP-α, and PDGFR etc^4–6^. Myofibroblasts in tumor are generally referred as tumor-associated fibroblasts or cancer-associated fibroblasts (CAF) and contribute as key fibro genic cells aggravating non-small cell lung cancer (NSCLC) growth and progression^7,8^. Thus, intervening myofibroblast-induced pro-fibrotic and pro-tumorigenic activities using signaling inhibitor can be an interesting approach against fibrosis and cancer.

Among multiple intracellular signaling pathways, activation of PI3K/Akt/mTOR pathway has been demonstrated to enhance inflammation, pro-survival activation in tumor cells, and deposition of ECM component^9^. A recent study demonstrated comparatively higher level of total and phosphorylated forms of Rapamycin (mTOR) in myofibroblasts^10^. Investigation on several mTOR inhibitors suggested that PI3K/Akt/mTOR pathway might be a potential target for anti-fibrosis therapy^11^. Besides, aberrant expression of histone deacetylases (HDACs) is frequent in human cancers and may participate in the progression of fibrotic development in idiopathic pulmonary fibrosis (IPS) and tumor progression^12–14^. Since HDAC inhibitors were widely exploited for their anti-fibrosis effects on liver/kidney fibrosis and their anti-cancer roles^15,16^, they may become other promising therapeutic drugs for lung and tumor fibrosis^17^.

Hence, CUDC-907, a newly-synthesized small molecular compound that has been designed by incorporating HDAC inhibitory functionality into a PI3K inhibitor pharmacophore comes into our sight^18,19^, while its impacts on fibrosis are not clear. It was reported that CUDC-907 exerts a potent anti-tumor activity against B cell lymphoma and other tumor cell lines^18^. Recently, a phase-I clinical trial on its biosafety, tolerability, and preliminary activity revealed its bright future in clinical application^20^.

This study investigated the anti-fibrotic effect of CUDC-907 using in vitro cell culture, in vivo xenograft model, which demonstrated that CUDC-907 could inhibit the proliferation, migration, invasion, and ECM deposition of in vitro cultured myofibroblasts and could also suppress collagen accumulation and tumor growth in vivo.

## Materials and methods

### Cell lines, cell culture, reagents, and CAF isolation

The fibroblast cell line NIH3T3 and human lung fibroblasts were purchased from Cell Bank of the Chinese Academy of Sciences (Shanghai, China) where they were characterized by mycoplasma detection, DNA –fingerprinting, isozyme detection, and cell vitality detection. These cell lines were immediately expanded and frozen such that they could be restarted every 6 months from a frozen vial of the same batch of cells. CUDC-907, GDC-0941 (PI3K/Akt/mTOR inhibitor), and Trichostatin A (HDAC inhibitor) solid powder was purchased from Selleck Chemicals (Houston, TX, USA). The compound powder was dissolved in pure dimethyl sulfoxide (DMSO) to make a concentration of 10 mM for storing under condition at 4 °C. Before using, the CUDC-907 stock solution was diluted in Dulbecco’s modified eagle medium (DMEM, Hyclone, Logan City, UT, USA) to result in designed drug concentrations with DMSO volume identical to that of vehicle control group. The final concentration of DMSO solvent was controlled under 0.001% (*v/v*) during each of following assays.

The cell lines were maintained in DMEM with 4.5 g/L of glucose, 2 mmol/L of L-glutamine, and 110 mg/L of sodium pyruvate, supplemented with 10% fetal bovine serum (FBS), and Antibiotic-Antimycotic (100×, Gibco, Grand Island, NY, USA) in a standard humidified incubator at 37 °C in 5% CO_2_ and 95% O_2_ atmosphere.

Human lung tissue samples were donated by 2 NSCLC patients (Table 1, Supplementary Fig. 1) and were collected according to a clinical protocol at Ruijin Hospital. This protocol was approved by the Ethics Committee of Ruijin Hospital (KY 2018-104). All patients were enrolled after written informed consent. Tissue samples were stored at −80 °C until their use for experiments.

**Table 1:** Characteristic of patients.

	Sex	Age	TNM stage	Pathological type
CAF1	Female	72	T1bN1M0	Adenocarcinoma
CAF2	Male	64	T2N1M0	Squamous cell carcinoma

Isolation and culture of fibroblasts were performed according to a previous protocol^21^. In brief, lung tissue samples were collected in sterilized 10-ml centrifuge tubes after surgical excision and then immersed and washed in HANK’s solution for 3 times with 5 min each time. Sterilized scalpels and scissors were used to mince the tissue into small pieces. These small pieces were treated with sufficient 0.25% collagenase (dissolved in Hank’s balanced salt solution, Gibco, Grand Island, NY, USA) for 1 h at 37 °C on a shaker. After digestion, cells were collected via centrifugation at 1500 RPM for 5 min and resuspended in DMEM supplemented with 10% fetal bovine serum (FBS, Hyclone) and Antibiotic-Antimycotic. Cells were seeded on a 10-cm culture dish at a density of 2 × 10^4^/cm^2^ and cultured in a 37 °C thermostat incubator containing 5% CO_2_ atmosphere. Cells were passaged at the same density before they reached 90% of confluence. The second passage cells were used in this research.

### CCK-8 cell proliferation analysis

Cell proliferation experiments were performed using the cell counting kit-8 (CCK-8; Dojindo, Japan). Cells were seeded at a density of 3000 cells/100 µL in five copies in 96-well opaque plates with clear bottoms (Falcon). The next day, cells were supplemented with 100 µL of fresh medium containing the indicated concentrations of CUDC-907 with or without 5 ng/mL TGFβ1. Same procedure was used when treating cells with 30 nM CUDC-907 or GDC-0941 or Trichostatin A, respectively. Medium with drug/vehicle was replenished every 48 h. CCK-8 solution was diluted in DMEM to obtain volume ratio as 1:10 before the test, and then the medium was replaced by 100 μL mixed liquid in each well tested. After incubation in a 37 °C thermostat incubator containing 5% CO_2_ atmosphere for 4 h, the medium was collected for measuring the optical density values at 450 nm using a microplate reader (Thermo Scientific, San Jose, CA, USA). Each assay was conducted and repeated for three independent pooled cell samples.

### Cell viability analysis

Live/Dead Viability/Cytotoxicity Kit (Invitrogen, Carlsbad, CA, USA) was used to evaluate cell viability. At day 3, the 96-well plates containing NIH3T3 cells treated with drug as shown above were incubated with a solution of 2 mM calcein-acetoxymethyl (Calcein-AM, R&D, Minneapolis, Canada) and 1 µg/ml propidium iodide (PI, Sigma-Aldrich, USA) for 30 min. Then reagents were washed out by PBS and plates were taken for phase contrast microscopy for assessment of gross features. In the Live/Dead assay, calcein-AM (GREEN) is a fluorescent dye that is activated by intracellular esterases, which is trapped inside the intact membrane of live cells. PI is a red fluorescent dye, which is impermeable to an intact cell membrane.

### Cell cycle and apoptosis analysis

Cells were plated at a density of 2 × 10^5^ in a six-well plate and treated with vehicle or CUDC-907 (10 nM and 30 nM) with or without TGFβ1 for 48 h. Subsequently, cells were detached with 0.25% trypsin-EDTA (Gibco), centrifuged and resuspended in phosphate buffered saline (PBS) in a 1.5-ml Eppendorf tube followed by 3 washes in PBS.

To examine the cell cycle profiles, cells were subsequently centrifuged at 1500 RPM for 5 min and then resuspended and fixed in 1 mL ice-cold 70% ethanol for 24 h. Afterward, fixed cell samples were centrifuged and washed in PBS. Cell pellets were stained using a cell cycle analysis kit (7sea Phamatech, Shanghai, P.R. China) and incubated at 37 °C for 30 min. Flow cytometer (Beckman Coulter, CA, USA) combined with ModiFit LT v2.0 software was applied in flow cytometric analyses.

To examine apoptosis, cells were resuspended and stained using Alexa Fluor 488 Annexin V/PI Apoptosis Kit (ThermoFisher Scientific, USA) and incubated at room temperature for 15 min. Then cells were analyzed by flow cytometry and fluorescence emission was measured at 530 nm. Each assay was repeated in three independent cell samples.

### In vitro cell migration assay

Scratch assay was employed to analyze the suppressive effect of CUDC-907 on migratory ability of NIH3T3 cells and CAF1 and CAF2. After over 90% confluence in six-well plates, the cell monolayer was scratched using a 200-μl pipette tip and then cultured in serum-free medium with CUDC-907 (10 nM, and 30 nM with or without TGFβ1) for 24 h. Photographs were taken at 0 h, 24 h after scratching and blank areas were measured by Image-Pro Plus (Version 6.0, Media Cybernetics, Maryland, USA). Percentages of area filled by cells were calculated according to the formula below: Cell migration rate (%) = (Area0h –Area24h)/Area0h ×100%. Three replicates were set in each group and normalized to DMSO group.

Subsequently, a transwell system (Corning, 8-μm pore size) was utilized for migration assay. Briefly, 3 × 10^4^ starved CAF1 suspended in 150 μl serum-free medium with CUDC-907 (10 nM and 30 nM with or without TGFβ1, respectively) were seeded in the upper compartment of the Boyden chamber in a 24-well plate. Lower compartment was filled with 500 μl DMEM with 10% FBS or pre-seeded with lung cancer cell lines, respectively. After 24-h incubation, a cotton swab was used to wipe non-migrated cells up on the top surface of the membrane, and the migrated cells on bottom surface was fixed in 4% paraformaldehyde and stained with 4′,6-diamidino-2-phenylindole (DAPI, Sigma, USA). The migrated cells in five randomly selected high-power fields were counted using Image-Pro Plus. This assay was repeated for three pooled cell samples.

### RNA extraction and complementary DNA synthesis

Total RNA was extracted from cells using Trizol reagent (Invitrogen Life Technologies Inc., NY, USA). RNA purity and concentration were confirmed via DU800 spectrophotometer (Beckman Coulter). cDNA was synthesized using AMV reverse transcriptase (Promega, WI, USA) with 2 μg of total RNA according to the manufacturer’s instruction as previously described^22^^22^

### Real-time quantitative polymerase chain reaction

Gene expression level was evaluated by quantitative real-time PCR (qPCR) analysis with Power SYBR Green PCR master mix (2×) (Applied Biosystems, Foster City, CA, USA). qPCR was conducted in a real-time thermal cycler (Stratagene Mx3000PTM QPCR System, La Jolla, CA, USA) according to the protocol published^22^. qPCR was performed as follows: 95 °C for 10 min then 40 cycles (95 °C for 30 s, 58 °C annealing temperature for 30 s and 72 °C for 45 s). Human housekeeping gene *GAPDH* was employed as an internal control. Each assay was performed in triplicate and all experiments were repeated in at least three different cell samples. The human primers for real-time qPCR analysis and their annealing temperatures are displayed in Table 2.

**Table 2:** Primers used in quantitative PCR analysis.

Gene	Primer Sequence (5′-3′)	Annealing temperature (°C)	Product size (bp)
Collagen I	Sense: GGCGGCCAGGGCTCCGACCC Antisense: AATTCCTGGTCTGGGGCACC	58	347
α-SMA	Sense: CATCATGCGTCTGGATCTGG Antisense: GGACAATCTCACGCTCAGCA	58	107
TGFβ1	Sense: AAGGACCTCGGCTGGAAGTG Antisense: CCGGGTTATGCTGGTTGTA	58	136
Collagen III	Sense: TGGTGTTGGAGCCGCTGCCA Antisense: CTCAGCACTAGAATCTGTCC	58	373

### Western blotting and p-Akt ELISA assay

Total protein was extracted from CAF1 treated as mentioned above by using RIPA lysis buffer plus 1% PMSF and 1% protein phosphatase inhibitor. Cell lysates were resolved on SDS-PAGE gels and then transferred to PVDF membranes. Immunoblotting was performed using standard procedures published^23^. The membranes were probed with the following primary antibodies against: Fibronectin (ab32419, Abcam, USA), Collagen III(ab184993, Abcam, USA), FAP(#66562, Cell signaling Technology, USA), BMP4(ab39973, Abcam, USA), PDGFR(#3174, Cell signaling Technology, USA), PDK1(#5662, Cell signaling Technology, USA), AKT(#4691, Cell signaling Technology, USA), p-AKT(Ser473)(#4060, Cell signaling Technology, USA), mTOR(#2983, Cell signaling Technology, USA), p-p70S6(T389)(#9234, Cell signaling Technology, USA), Smad2/3(#8685, Cell signaling Technology, USA), HDAC1-6(#5356, #5113, #3949, #3443, #7558, Cell signaling Technology, USA), Acetyl-Histone H3(#4243, Cell signaling Technology, USA),β-actin(#4970, Cell signaling Technology, USA). Then, the membranes were subsequently incubated with secondary antibody (Anti-rabbit IgG #7074, Anti-mouse IgG, #7076, Cell signaling Technology, USA) coupled with Horseradish Peroxidase (HRP). Enhanced Chemiluminescence (ECL) detection was applied to visualize the protein band.

To further understand the effect of CUDC-907 in regulating Akt phosphorylation, CAF1 were seeded at a density of 2 × 10^6^ in a 9 cm^2^ dish plate and treated with vehicle or CUDC-907 (10 nM and 30 nM) with or without TGFβ1 and collected at different time points (4 h, 8 h, 12 h, 24 h, 48 h). p-Akt levels of cell lysates were analyzed by using a Akt(Phospho) [pT308] Multispecies InstantOne ELISA kit(#85-86044-11, Thermofisher science, USA).

### Animal studies

C57BL/6 male mice and nude male mice aging from 6 to 8 weeks were purchased from Shanghai Slaccas experimental animal limited liability company. All animal experimental protocols were approved by the Animal Care & Welfare Committee of Shanghai ninth people’s hospital affiliated to Shanghai Jiaotong University School of medicine. C57BL/6 mice were assigned to one of the following three treatment group: (1) controls receiving intratracheal instillation of NaCl 0.9% solution; (2) mice treated with intratracheal instillation of bleomycin(30 µL-2mg/kg) on day 0 and then treated as a control with 30% Captisol by oral gavage; (3) mice treated with bleomycin on day 0 and then treated as a treatment group from day 3, which was administered 50 mg/kg CUDC-907(resolved in 30% Captisol) by oral gavage, on a 5-day on/ 2-day off dosing regimen. Body weight was measured weekly and mice were sacrificed at day 18. The lungs were lavaged, and then snap-frozen in liquid nitrogen for hydroxyproline assay, or 4% paraformaldehyde fixed, paraffin embedded, for immunohistochemistry.

To verify the effect of CUDC-907 in regulating CAF in lung cancer, xenograft model was used. A549 cell line and CAF1 expressing luciferase (3:2) mixtures were injected subcutaneously into axilla of mice to establish the NSCLC model. After tumor formation (Day 10), mice were divided randomly into two groups: control, CUDC-907 treatment. The mice in control group were treated with 30% Captisol and mice in treatment group were treated with 50 mg/kg CUDC-907 (resolved in 30% Captisol) by oral gavage, on a 5-day on/ 2-day off dosing regimen. Fluorescein is a compound for bioluminescence imaging (BLI). At Day 10, 25, and 31, mice were anesthetized with isoflurane and then injected with 75 mg/kg D-luciferin solution (MCE, Monmouth Junction, USA) for imaging. The photos of mice were obtained from Image Studio Instrument (Lincoln, Nebraska, USA). Body weight and tumors were measured every three days. After euthanasia, primary tumor tissues were snap-frozen and some primary tumors were 4% paraformaldehyde fixed, paraffin embedded, and sectioned for immunohistochemistry. No blinding was done for animal studies.

### Histologic analysis

Left lung and tumor sections were stained with hematoxylin and eosin (HE) and Masson’s trichrome staining was performed to assess the formation of lung collagen. The whole section was imaged with a NIKON ECLIPSE C1 microscope and tiled using 10% image overlap into a single panoramic by NIKON DS-U3 software (NIKON).

### Hydroxyproline assay

Lung collagen content was evaluated using hydroxyproline assay. Briefly, tissue was minced and hydrolyzed in 1 ml 12 N HCl at 100 °C for 20 min, the hydroxyproline was detected by incubation with chloramine T and p-dimethylaminobenzaldehyde, and the absorbance was measured at 550 nm. Each sample was run in triplicate. Collagen content in lung was expressed as micrograms of collagen per lung and was converted from micrograms of hydroxyproline.

### Immunohistochemical and immunofluorescence staining

Lung and tumor specimens were fixed in 4% paraformaldehyde at 4 °C overnight and then embedded in paraffin. Sections were deparaffinized, serially rehydrated, treated with 1×citrate buffer at 120 °C for antigen retrieval, blocked, and then immunostained with antibodies (Information showed in Western blotting) over night at 4 °C. 3,3′-diaminobenzidine (DAB, Servicebio, G1211) with GTVision^TM^ III detection system/Mo&Rb (Dako, K5007) was used to detect immunoreactivity of the primary antibody. Slides were scanned at 20× magnification using CICXSP-C204 microscope (NIKON).

Paraffin embedded sections were also stained with α-SMA antibody and IgG isotype controls. Where indicated in the figure legends, mosaic images were generated from multiple ×20 images captured on a NIKON ECLIPSE C1upright fluorescent microscope and tiled using 10% image overlap by NIKON DS-U3 software (NIKON).

### Statistical analysis

All experiments were repeated at least three times. All the data were presented as mean ± standard deviation (SD). Unpaired *t* test One-Way ANOVA was used to judge if there was a significant difference among multiple groups, and subsequently *p* values were calculated by LSD test. All statistical calculations were conducted via the software SPSS (version 22.0, SPSS Inc., IL, USA). Difference was considered significant when *p* values were <0.05.

## Results

### CUDC-907 inhibits cellular proliferation and causes G1/S arrest and apoptosis

**Fig. 1:**
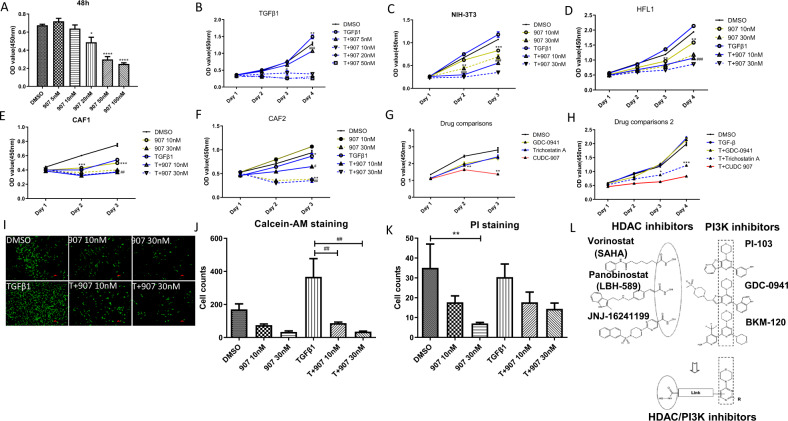
CUDC-907 inhibits TGFβ1 induced fibroblasts proliferation. The antiproliferative effect of CUDC-907 was tested in NIH-3T3 cell line, Human fetal lung fibroblasts (HLF1) and two primary cancer-associated fibroblasts (CAF1 & CAF2) with or without TGFβ1 treatment by CCK-8 assay. OD value was measured at length of 592 nm. **a** NIH-3T3 cells were treated with CUDC-907 in concentration gradient for 48 h. **b** TGFβ1-stimulated NIH-3T3 cells were treated with CUDC-907 at dose ranging from 5 to 50 nM. **c**–**f** Fibroblasts cell lines stimulated with or without TGFβ1 were treated with CUDC-907 10 nM and 30 nM for 3 days, respectively. **g** CAF1 were treated with 30 nM GDC-0941, Trichostatin A, and CUDC-907, respectively. **h** HLF1 stimulated with TGFβ1 were treated with CUDC-907. **i** Fluorescence microscopic images of NIH-3T3 cells stimulated with or without TGFβ1 72 h post treatment with 10/30 nM CUDC-907 and exposed to Calcein AM fluorescent dye. Scale bars: 100 µm. **j** Cell numbers were counted and analyzed from Calcein AM staining images. **k** Cell numbers were count in PI staining images (Images were not shown). Error bars are ±SD. *, **, *** represent 907 treatment groups compared with DMSO group, respectively. **l** Chemical structure of CUDC-907. *P < 0.05; **P < 0.01; ***P < 0.001. #, ##, ### represent T + 907 treatment groups compared with TGFβ1 group, respectively. ^#^*P* < 0.05; ^##^*P* < 0.01; ^###^*P* < 0.001.

We first investigated the effect of CUDC-907 treatment on cellular proliferation using 4 different fibroblast origin: 2 fibroblast cell lines and 2 primary lung CAF from squamous and adenocarcinomas [Table 1, Supplementary Fig. 1]. We found that CUDC-907 treatment significantly inhibited cellular proliferation in a time- and dose-dependent manner and caused cell death at higher concentrations regardless of TGFβ1 stimulation (Fig. 1a–f). These results were validated by Calcein AM/PI fluorescent staining (Fig. 1i–k). Furthermore, we compared the anti-proliferate effect of CUDC-907 with PI3K inhibitor GDC0941 and HDAC inhibitor Trichostatin A for CUDC-907 was designed based on their active ingredients(Fig. 1l)^19^. We found that CUDC-907 showed higher efficiency in inhibiting fibroblasts proliferation than GDC0941 and Trichostatin A at the same dose.

**Fig. 2:**
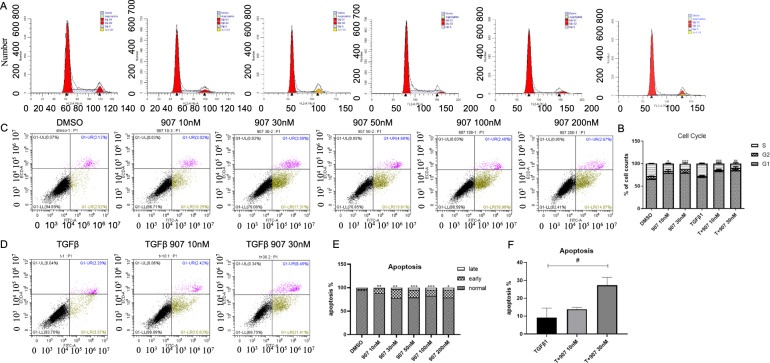
CUDC-907 treatment causes G1-S arrest and induces apoptosis. **a** HLF1 stimulated with or without TGFβ1 were treated with 10 and 30 nM of CUDC-907 for 12 h. Cells were then stained with PI and analyzed by FACS. CUDC-907 caused a significant increase in the G1-S peak in all cell lines tested at the 12-h time-point after drug treatment. **b** Histogram representation of the percentage of cells in different cell-cycle phases. **c** HLF1 were treated with CUDC-907 at dose ranging from 10 to 200 nM. Cells were then stained with Annexin V/PI and analyzed by FACS. **d** HLF1 stimulated with TGFβ1 were treated with 10 and 30 nM of CUDC-907. Cells were then stained with Annexin V/PI and analyzed by FACS. **e** Histogram representation of the percentage of cells in different apoptosis phases in **c**. **f** Histogram representation of the percentage of apoptosis cells in **d**. Error bars are ±SD. *, **, *** represent 907 treatment groups compared with DMSO group, respectively. *P < 0.05; **P < 0.01; ***P < 0.001. #, ##, ### represent T + 907 treatment groups compared with TGFβ1 group, respectively. ^#^*P* < 0.05; ^##^*P* < 0.01; ^###^*P* < 0.001.

To understand the mechanism by which CUDC-907 treatment inhibited cellular proliferation, we tested it effect on cell cycle progression and apoptosis. We treated NIH-3T3 and HLF1 cell lines with CUDC-907 for 24 h and observed G1-S arrest post drug treatment (Fig. 2a, b and Supplementary Fig. 2).

We next evaluated the effect of CUDC-907 treatment on apoptosis using the Annexin V/PI assay and found increased activity at 24 h that was dose dependent (Fig. 2d, f). To further determine the effect of CUDC-907 treatment in regulating myofibroblasts, we stimulated HLF1 with TGFβ1 and subsequently added CUDC-907. We found that CUDC-907 treatment significantly increased apoptosis in a dose-dependent manner (Fig. 2e, g).

### CUDC-907 inhibits cell migration and invasion

**Fig. 3:**
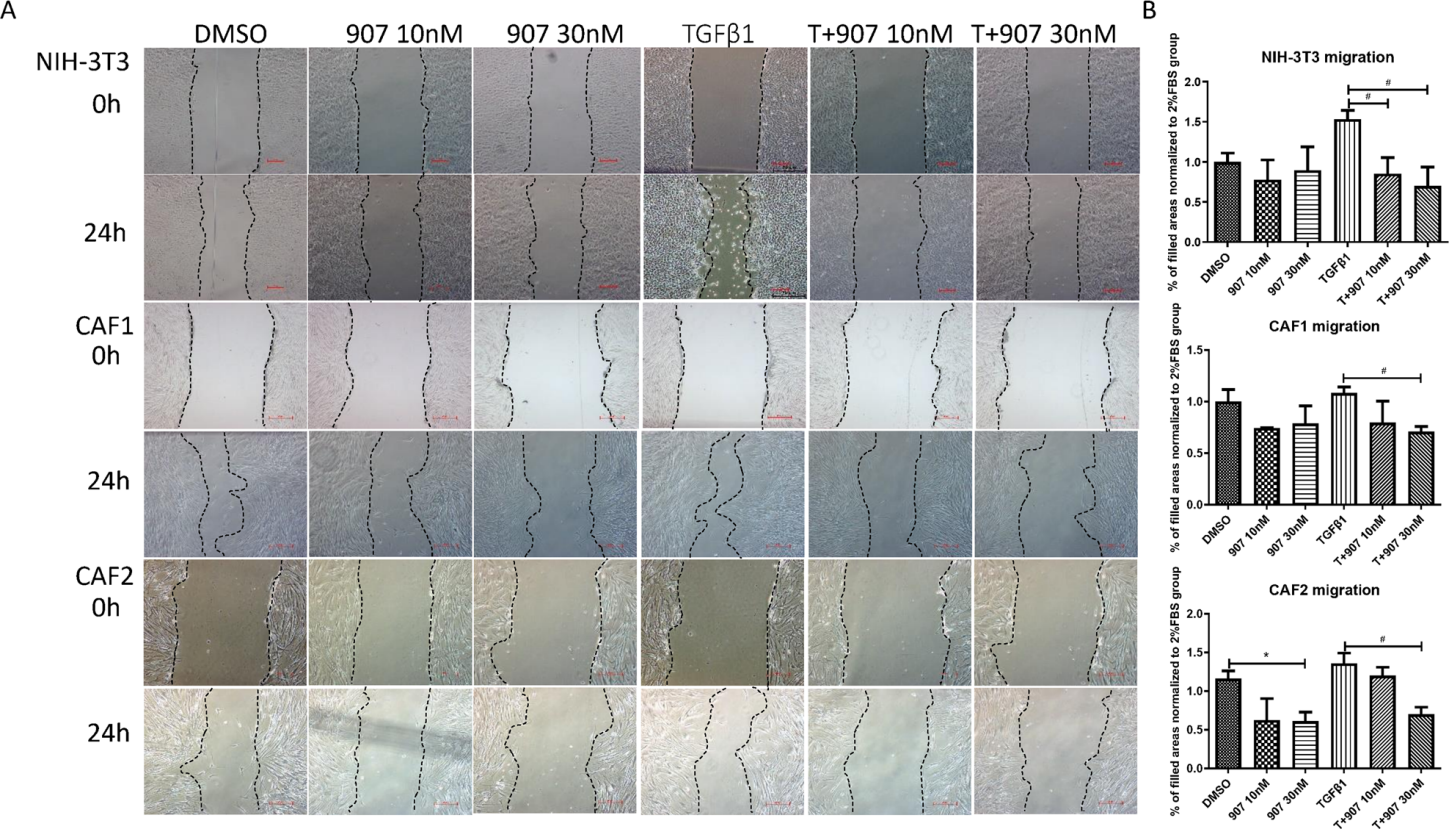
CUDC-907 inhibits TGFβ1 induced fibroblasts migration. **a** Live-cell images acquired 24 h after scratching in a wound-healing assay to compare effects of 10 nM and 30 nM CUDC-907 in Fibroblasts cell lines stimulated with or without TGFβ1. Scale bars: 50 µm. **b** Cell migration is quantitated by measuring gap width variation of A and analyzed by Image. Error bars are ±SD. *, **, *** represent 907 treatment groups compared with DMSO group, respectively. *P < 0.05; **P < 0.01; ***P < 0.001. #, ##, ### represent T + 907 treatment groups compared with TGFβ1 group, respectively. ^#^*P* < 0.05; ^##^*P* < 0.01; ^###^*P* < 0.001.

**Fig. 4:**
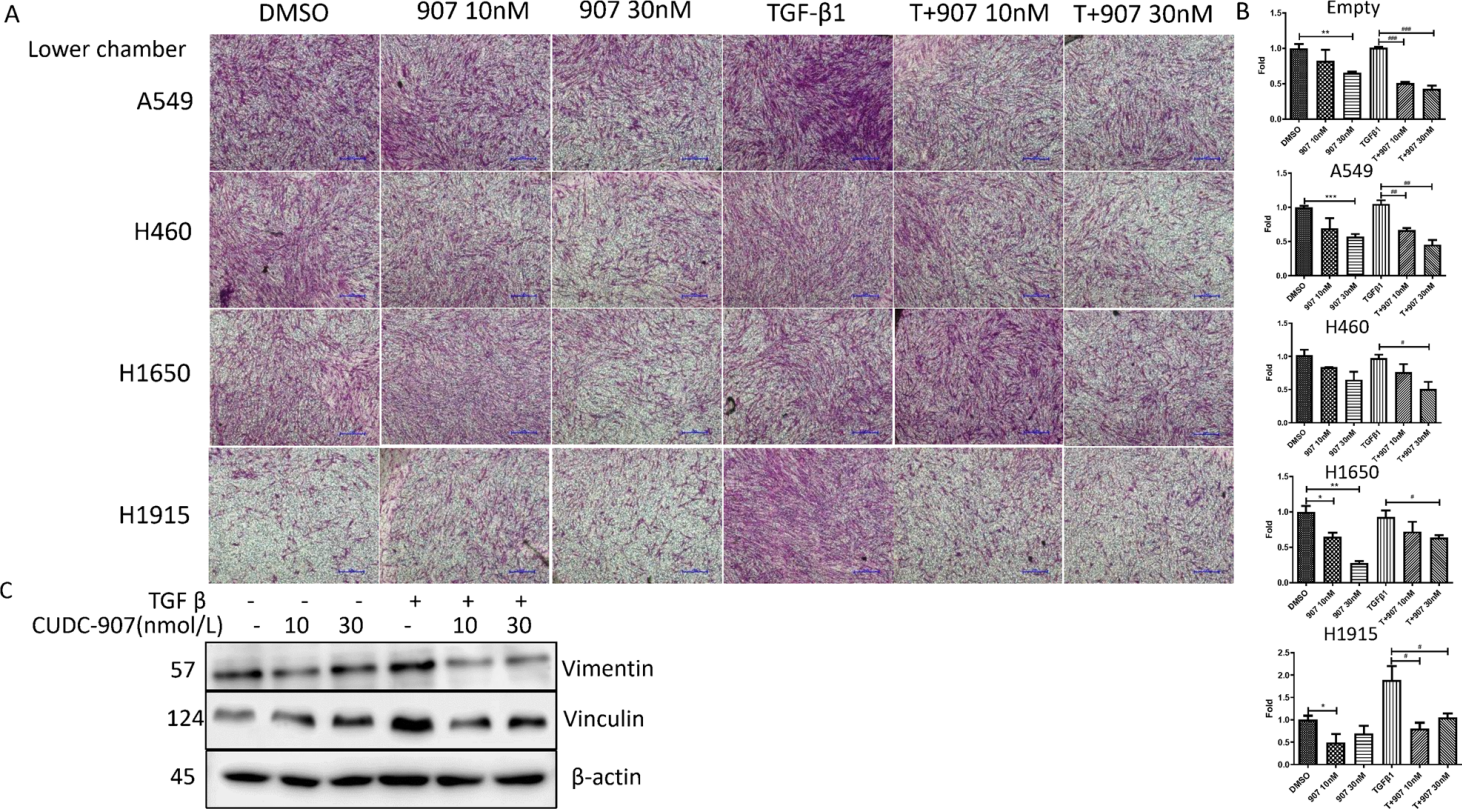
CUDC-907 treatment inhibits lung cancer cell lines induced migration with or without TGFβ1 stimulation. **a** Migration assays performed with TGFβ1 stimulated CAF1 treated with 10 nM and 30 nM CUDC-907 and cocultured with lung cancer cell. After incubation for 24 h, cells that migrated through transwell membrane were fixed, stained and acquired. Scale bars: 25 µm. **b** Representative micrographs and graphic representation of the quantification of three independent experiments each using five random fields. **c** Western blot analysis of vimentin and vinculin. Error bars are ±SD. *, **, *** represent 907 treatment groups compared with DMSO group, respectively. *P < 0.05; **P < 0.01; ***P < 0.001. #, ##, ### represent T + 907 treatment groups compared with TGFβ1 group, respectively. ^#^*P* < 0.05; ^##^*P* < 0.01; ^###^*P* < 0.001.

One of the hallmarks of fibroblasts is their ability to respond to TGFβ1 and become activated, resulting in enhanced properties of proliferation, migration and production of growth factors and extracellular matrix (ECM) which is the biology and function of fibroblasts in cancer In order to investigate the suppressive effect of CUDC-907 on myofibroblasts migration capacity, in vitro scratched assay and migration assay were employed. As shown, at 24 h post scratching, CUDC-907 treatment significantly decreased cellular migration and invasion in all four fibroblast sources with or without TGFβ1 stimulation (Fig. 3a, b). We next evaluated the effect of CUDC-907 treatment on CAF1 cocultured with lung cancer cell lines. In the transwell migration assay, four lung cancer cell lines were seeded in down chamber and migrated cells decreased in a dose-dependent manner with CUDC-907 treatment (Fig. 4a, b).

We further evaluated the effect of CUDC-907 treatment on proteins known to regulate fibroblasts migration and invasion. Vimentin is recognized as a major type III intermediate filament protein participates in cell adhesion, migration and invasion^24^. Another protein is Vinculin that is present in cell–cell junctions and plays a key role not only in the generation of traction forces, but also in directional migration of cells^25,26^. We found that the protein expression of Vimentin was reduced with CUDC-907 treatment, but there was a modest compensatory increase in vinculin protein levels in CAF1 (Fig. 4e).

### CUDC-907 attenuated TGFβ1-induced fibroblast differentiation and collagen expression

In lung cancer fibrosis, persistent emergence and accumulation of cancer cells in a given tissue represents an ongoing tissue injury, initiating a chronic wound healing response towards the cancer cells. CAF are largely governed by the growth factors released by cancer cells. Among these factors, TGFβ is one of the most important key mediators of CAF activation.

**Fig. 5:**
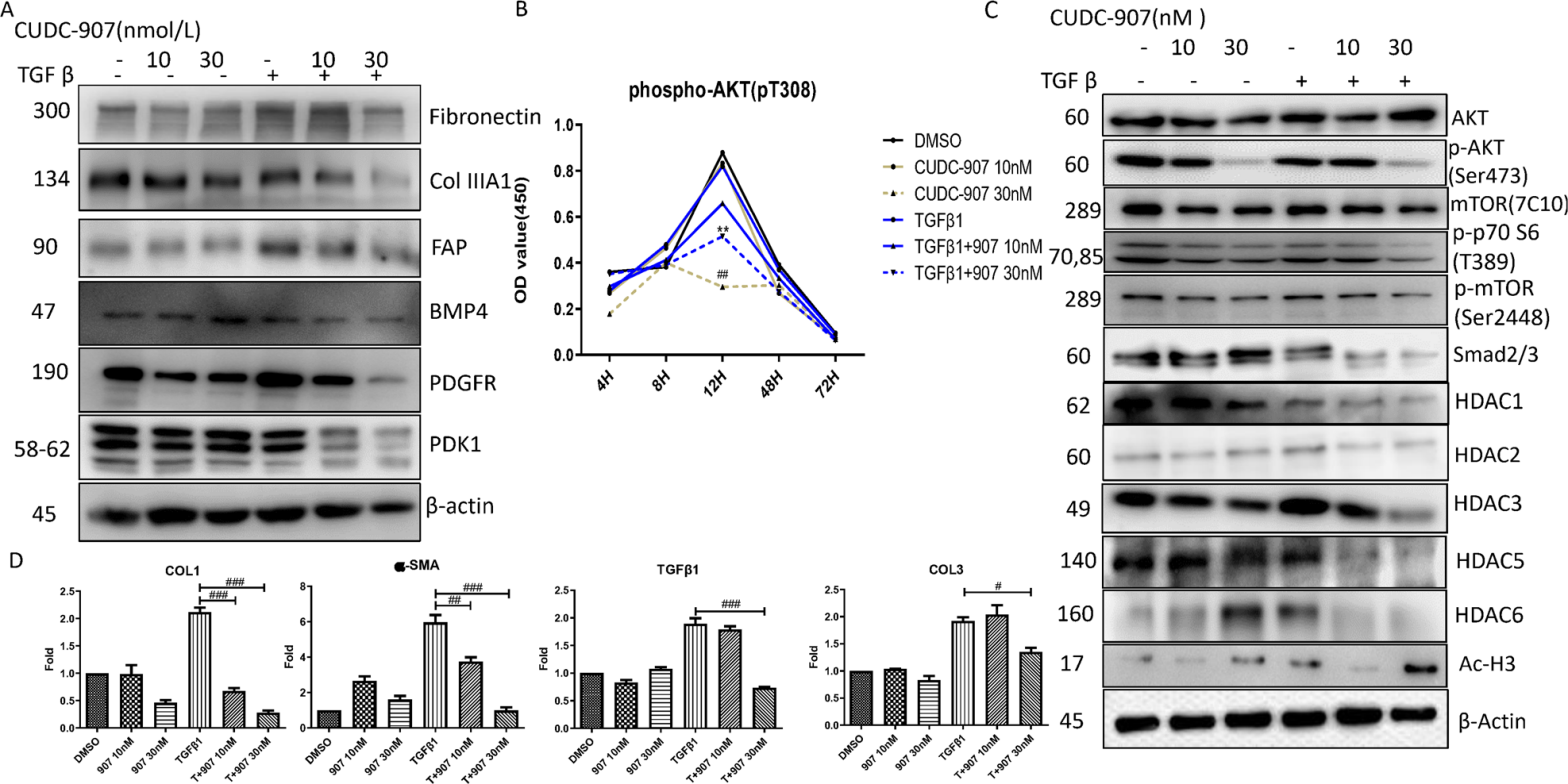
CUDC-907 inhibits TGFβ1 induced fibrosis relative proteins and mRNA expression levels. **a** Western blot analysis of fibrosis relative proteins and myofibroblasts markers. CAF1 were stimulated with TGFβ1 or left unstimulated for 48 h in the presence or absence of 30 or 50 nM CUDC-907. **b** Western blot analysis of PI3K downstream proteins and HDACs. Full length blots were presented in Supplementary Fig. 3 and 4. **c** ELISA analysis was performed to detect p-akt expression in different time points (4 h, 8 h, 12 h, 48 h, 96 h) after CUDC-907 treatment. **d** qPCR results from CAF1 received the same treatment as described in A. Error bars are ±SD. *, **, *** represent 907 treatment groups compared with DMSO group, respectively. *P < 0.05; **P < 0.01; ***P < 0.001. #, ##, ### represent T + 907 treatment groups compared with TGFβ1 group, respectively. ^#^*P* < 0.05; ^##^*P* < 0.01; ^###^*P* < 0.001.

To test antifibrotic activity of CUDC-907, we evaluated whether CUDC-907 treatment influenced CAF differentiation and collagen expression under TGFβ stimulation. CUDC-907 treatment significantly inhibited collagen, fibronectin, PDGFR, and PDK1 (Fig. 5a, d). We next evaluated the effect of CUDC-907 treatment on proteins known to regulate CAF differentiation. We found that protein expression of FAP, PDGFR, and PDK1 was decreased, especially at the concentration of 30 nM (Fig. 5a). To understand the mechanism of CUDC-907 in regulating TGFβ-induced tumor fibrosis, we evaluated PI3K/AKT signaling protein levels of TGFβ1-stimulated CAF1 treated with CUDC-907. As expected, we found that CUDC-907 treatment effectively reduced phospho-AKT, phosphor-p70S6, phosphor-mTOR, and Smad2/3levels (Fig. 5b, c).

We also evaluated the effect of CUDC-907 as a histone deacetylase inhibitor in CAF1 and the specific HDAC it targets. CUDC-907 treatment in CAF1 resulted in increased acetylation of histone 3, consistent with its effect as a histone deacetylase inhibitor (Fig. 5c). However, the effect of CUDC-907 on specific HDAC protein levels is unknown. Because HDAC1, HDAC2, HDAC3, HDAC5and HDAC6 have been reported to be overexpressed in CAF, we evaluated the effect of CUDC-907 treatment on these specific HDAC proteins. We found that CUDC-907 treatment reduced TGFβ1-induced HDAC1, HDAC2, HDAC3, HDAC5, and HDAC6 (Fig. 5c).

### CUDC-907 attenuated collagen expression and production of in bleomycin-induced lung fibrosis in mice

**Fig. 6:**
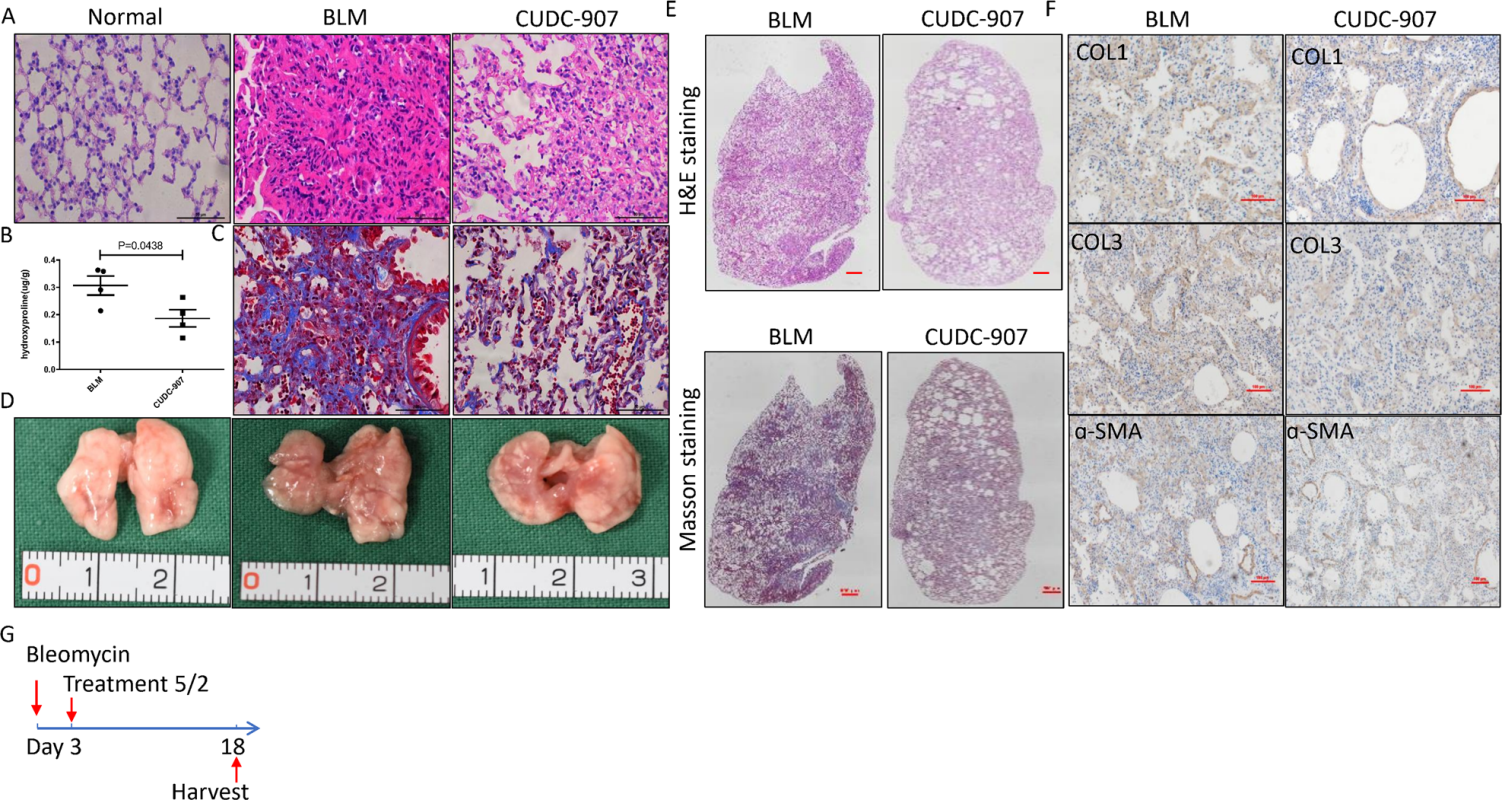
CUDC-907 attenuates bleomycin induced mice fibrosis. **a** HE staining of lung sections from Vehicle, vehicle and CUDC-907 chow mice 18 days after bleomycin. Scale bars: 50 µm. **b** Hydroxyproline analysis of lung sections from mice given bleomycin saline chow (*n* = 4) or bleomycin CUDC-907 chow (*n* = 4). **c** Masson’s trichrome staining of lung sections from Vehicle or CUDC-907 treated mice 7 days after bleomycin. **d** Representative pictures of lung sections treated with Vehicle or CUDC-907 chow. **e** Mosaic images (×4) covering whole lung section from **a** and **c** are shown. **f** IHC for Collagen I, Collagen 3 and α-SMA in Vehicle or CUDC-907 chow mice 18 days after bleomycin. Representative pictures are acquired. Scale bars: 100 µm. Error bars are ±SD.

Whether CUDC-907 could attenuate ECM deposition is essential for assessing its anti-fibrosis effect. Thus, we evaluated the in vivo effect of clinical-grade CUDC-907 in a fibrosis mouse model that recapitulates the clinical behavior of idiopathic pulmonary fibrosis. Mice were treated by either 35% Captisol (control) or 1 mg/kg CUDC-907 dissolved in 35% Captisol after intratracheal bleomycin injection (Fig. 6g). We found that CUDC-907 treatment inhibited collagen accumulation (Fig. 6a, c, d). At day 18 these mice exhibited marked attenuation of bleomycin-induced total lung collagen deposition (Fig. 6b). Furthermore, immunohistochemical staining of left lung sections showed that CUDC-907 treatment inhibits bleomycin-induced total Col1, Col3, and ɑ-SMA (Fig. 6f).

### CUDC-907 inhibits tumor growth and fibrosis in a human xenograft mouse model constituting both CAF1 and A549 cell line

**Fig. 7:**
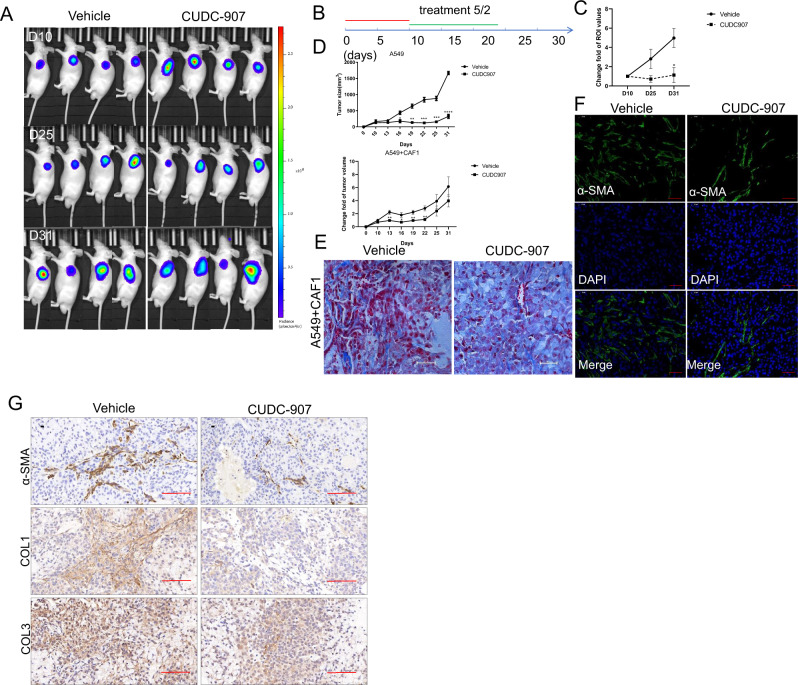
CUDC-907 treatment inhibits tumor growth and fibrosis. **a** Mixed A549 and CAF1 transduced with D-luciferase were injected subcutaneously into NOD/SCID mice and in vivo bioluminescent signal was quantified before and after CUDC-907 treatment. Representative in vivo images of PBS or CUDC-907 treated (5/2, 50 mg/kg) mice are acquired. **b** treatment schema. Mice were divided into 2 groups: Vehicle and treatment (*n* = 5 in each group) and injected with A549 cells or mixed A549 and CAF1(3:2). Treatment with CUDC-907 was started on day 10. CUDC-907 was administered by oral gavage (5/2, 50 mg/kg). **c** Tumor-to-background ratios of fluorescence intensities (*n* = 5). CUDC-907 treatment significantly decreased tumor fibrosis as measured by whole-body luciferase activity. **d** CUDC-907 treatment significantly decreases tumor growth with or without fibroblasts mixture. **e** Masson’s trichrome staining of tumor sections from Vehicle or CUDC-907 treated mice 10 days after subcutaneous injection of mixed A549 and CAF1. Scale bars: 100 µm. **f** Representative IF staining pictures in Vehicle and treated group mice. Scale bars: 25 µm. **g** IHC for Collagen 3 and α-SMA in Vehicle or CUDC-907 chow mice. Representative pictures are acquired. Scale bars: 100 µm. Error bars are ±SD. *, **, *** represents 907 treatment group compared with vehicle group. *P < 0.05; **P < 0.01; ***P < 0.001.

The effectiveness of CUDC-907 was also tested in a classic xenograft model as illustrated in Fig. 7b by injecting A549 cells subcutaneously. In order to assess CUDC-907 treatment in inhibiting tumor fibrosis, we mixed CAF1 with A549 human adenocarcinoma cells in a 3:2 ratio and inoculated these mixtures subcutaneously in immune-deficient nude mice. Luciferase was induced into CAF1s to allow their detection in vivo.

At day 25 and day 31 after drug administration, CUDC-907 treatment resulted in significantly less overall tumor burden in both CAF1 presence and absent mouse models (Fig. 7a, d). Furthermore, numbers of CAF1 was significantly decreased in the CUDC-907 treatment group (Fig. 7c). Masson’s trichrome staining, IF and IHC showed significantly reduced collagen accumulation, as well as a-SMA and Collagen 3 expression (Fig. 7e, f, g).

## Discussion

In this study, we evaluated the antifibrosis activity of CUDC-907, a first-in-class dual PI3K, and HDAC inhibitor, in in vitro and in vivo studies. We found that CUDC-907 inhibits TGFβ1-induced fibroblasts proliferation, migration. Moreover, it effectively inhibits commonly activated signaling pathways in lung and tumor fibrosis, and induces G1-S arrest and apoptosis. We also found that HDAC2 is highly expressed after TGFβ1 stimulation and this effect could be prevented via CUDC-907 treatment. Thus, our data show that CUDC-907 is a promising candidate against fibrosis and tumor progression.

TGFβ1 has been shown to be a key modulator in the synthesis of ECM, and also stimulates various intracellular pathways that can promote cell viability. Although attractive as a target, the advantage of TGF β1 in suppressing inflammation and epithelial proliferation made it not a good idea of global inhibition of TGFβ1 signaling^27^. It has been reported that PI3K-AKT signaling pathways are commonly induced in myofibroblasts by TGFβ1 and targeting mTOR mediates many physiological functions such as metabolism, cell cycle progression, proliferation, migration, and also contributes to collagen accumulation^28^. PI3K activation results in production of membrane-localized phosphatidylinositol-3,4,5-trisphosphate (PIP_3_) and lead mTOR functions at two distinct nodes in this axis. mTOR complex1 (mTORC1) signaling induces canonical Smad activation via p70S6K and 4E-BP1 phosphorylation which strongly mediates the fibro genic effects of TGF β1. mTOR complex 2 (mTORC2) and 3-phosphoinositide-dependent protein kinase-1(PDK1) phosphorylate Akt (Ser473) and SGK1 that leads to human lung fibroblasts proliferation and differentiation^29,30^. The reduction of gene expression of *TGF-β1* and abrogation of its downstream cascade may, thus, elucidate why CUDC-907 showed such potent anti-fibrotic capacity^31^.

HDACs maintain a dynamic equilibrium in the cell in conjunction with histone acetyl transferases, which deacetylate lysine residues on histones to bring about transcriptional expression. TGFβ1-induced myofibroblasts activation was accompanied by a general reduction in histone acetyltransferases (HAT), and divergent changes in histone deacetylase (HDAC) enzymes at both transcript and protein levels^32^. The activities of all Class I and II HDACs were reported to be significantly upregulated in IPF lung tissues^33^. Many studies have used HDAC inhibitors to block lung fibrotic process and tumor growth, and SAHA, a nonselective HDAC Class I and II inhibitor, has been reported to have the ability to inhibit the differentiation of TGFβ1-induced myofibroblasts^34–36^. Similarly, Trichostatin A, a recently proved HDAC inhibitor, works at a micromolar level and sufficiently inhibits fibroblasts in vitro^17^. However, increased apoptosis resistance of myofibroblasts results in cellular resistance to certain HDACIs^33^. For example, HDAC2 is increased in the middle and late stages of bleomycin-induced lung fibrosis in mice^37^ and is associated with resistance to Fas-mediated apoptosis^38^. Thus, monotherapy with agents that target a driver event is not likely to yield durable responses. Thus, the effective inhibition of multiple pathways active in myofibroblasts with CUDC-907 treatment, i.e. its effect as an HDAC and PI3K inhibitor, suggest that it may be more effective than monotherapy.

So far it has been shown that suppression of histone deacetylase and phosphoinositide 3-kinase simultaneously worked better than single HDAC inhibitor for the treatment of cutaneous T-cell lymphoma or other cancers^39,40^. Besides, a hybrid molecule like CUDC-907 becomes difficult in escaping from cells after being converted into its pharmacologically-active acid form so that its cell concentration easily build-up^41^. Therefore, this newly-synthesized small molecular compound designed as a dual inhibitor of PI3K/Akt/mTOR and HDAC, came into our sight and was tested in this study. In our studies to understand the mechanism of action of CUDC-907 on cellular proliferation, migration, and invasion, and target HDACs, we have identified that 30 nM of CUDC-907 could potently suppress migration and invasion ability of TGF β1-induced fibroblasts and CAF as well as their ability of collagen production. Moreover, we compared the inhibitory effect of CUDC-907, GDC-0941, and Trichostatin A at 30 nM in this research and confirmed that CUDC-907 inhibited myofibroblasts better in cell proliferation, indicating that CUDC-907 may be a better option for lung fibrotic disease therapy.

Such similar phenomena were also reported in previous published investigations. Different anti-proliferative mechanisms of mTOR or HDAC inhibitor have been reported, such as G0-G1 and G2-M cell cycle arrest, or induced apoptosis^11,42–45^. Previous study revealed that CUDC-907 induced a G2/GM cell cycle arrest of H460, a human tumor cell line^19^, indicating that CUDC-907 may interfere in cell mitosis. We found that CUDC-907 causes cell-cycle arrest at G0/G1, followed by activation of apoptosis post treatment. This effect was also reported in human bone marrow stromal cells when exposed to CUDC-907^46^. Kotian et al.^23^ discovered that CUDC-907 induced G2-M arrest and apoptosis by downregulating cyclin B1, AURKA, AURKB, and PLK1 while upregulating p21 and p27 levels. However, the mechanism of CUDC-907 in regulating G0-G1 arrest is still unknown. Simultaneously, the attenuated ability of cell migration and invasion in myofibroblasts was relevant to the inhibition of PI3K pathway as well as the function of HDAC^47,48^. The anticancer effects of CUDC-907, such as attenuation of migration and invasion were realized partially by induction of TWIST1 and E-cadherin expression. However, the main mechanism remains to be explored.

As an oral, first-in-class and rationally-designed, compound, CUDC-907 was recently applied in a phase-I clinical trial of relapsed or refractory lymphoma or multiple myeloma, that revealed its potential value in future clinical application for cancer therapy^20^. Furthermore, the US FDA has granted its orphan drug designation for the treatment of patients with Diffuse Large B-cell Lymphoma. The role of CUDC-907 in targeting myofibroblasts revealed in the current study may suggest that CUDC-907 could potentially be used as a drug for fibrosis and cancer therapy with further confirming studies on clinical efficacy and biosafety.

In summary, we confirmed the efficient inhibition of both PI3K/Akt/mTOR and HDAC by CUDC-907, and therefore demonstrated its potent counteractive role in suppressing proliferation, collagen production and ECM deposition, and inhibiting the properties of migration and invasion of fibroblasts and CAF. All of these represent a therapeutic alternative strategy for lung fibrosis and cancer that should be investigated in a clinical future.

## Supplementary information

Supplementary Figure Legends

Supplementary Fig.1

Supplementary Fig.2

Supplementary Fig.3

Supplementary Fig.4
