# Supplementary material for: Dual inhibition of HDAC and tyrosine kinase signaling pathways with CUDC-907 attenuates TGFβ1 induced lung and tumor fibrosis
Source: Cell Death Dis. 2020 Sep 17;11(9):765. doi: 10.1038/s41419-020-02916-w (PMC7499263; doi:10.1038/s41419-020-02916-w)
Supplement: Supplementary file 1 — Supplementary Figure Legends [file 41419_2020_2916_MOESM1_ESM.docx]

Supplementary Figure Legends

Figure1. A. Representative images of patients’ CT scanning. B. Luminescence of CAF1 transduced with D-luciferase tested by seeding cells in a half form. Wells filled with medium were used as control.

Figure2. NIH3T3 stimulated with or without TGFβ1 were treated with 10 and 30nM of CUDC-907 for 12 hours. Cells were then stained with PI and analyzed by FACS. CUDC-907 caused a significant increase in the G1-S peak in all cell lines tested at the 12-hour time-point after drug treatment.

Figure3. Original files of part full length blots showed in Fig.5 were displayed and molecular weight of each blots were indicated.

Figure4. Original files of part full length blots showed in Fig.4 were displayed and molecular weight of each blots were indicated.
