# Supplementary figures and images for: Dual inhibition of HDAC and tyrosine kinase signaling pathways with CUDC-907 attenuates TGFβ1 induced lung and tumor fibrosis
Source: Cell Death Dis. 2020 Sep 17;11(9):765. doi: 10.1038/s41419-020-02916-w (PMC7499263; doi:10.1038/s41419-020-02916-w)

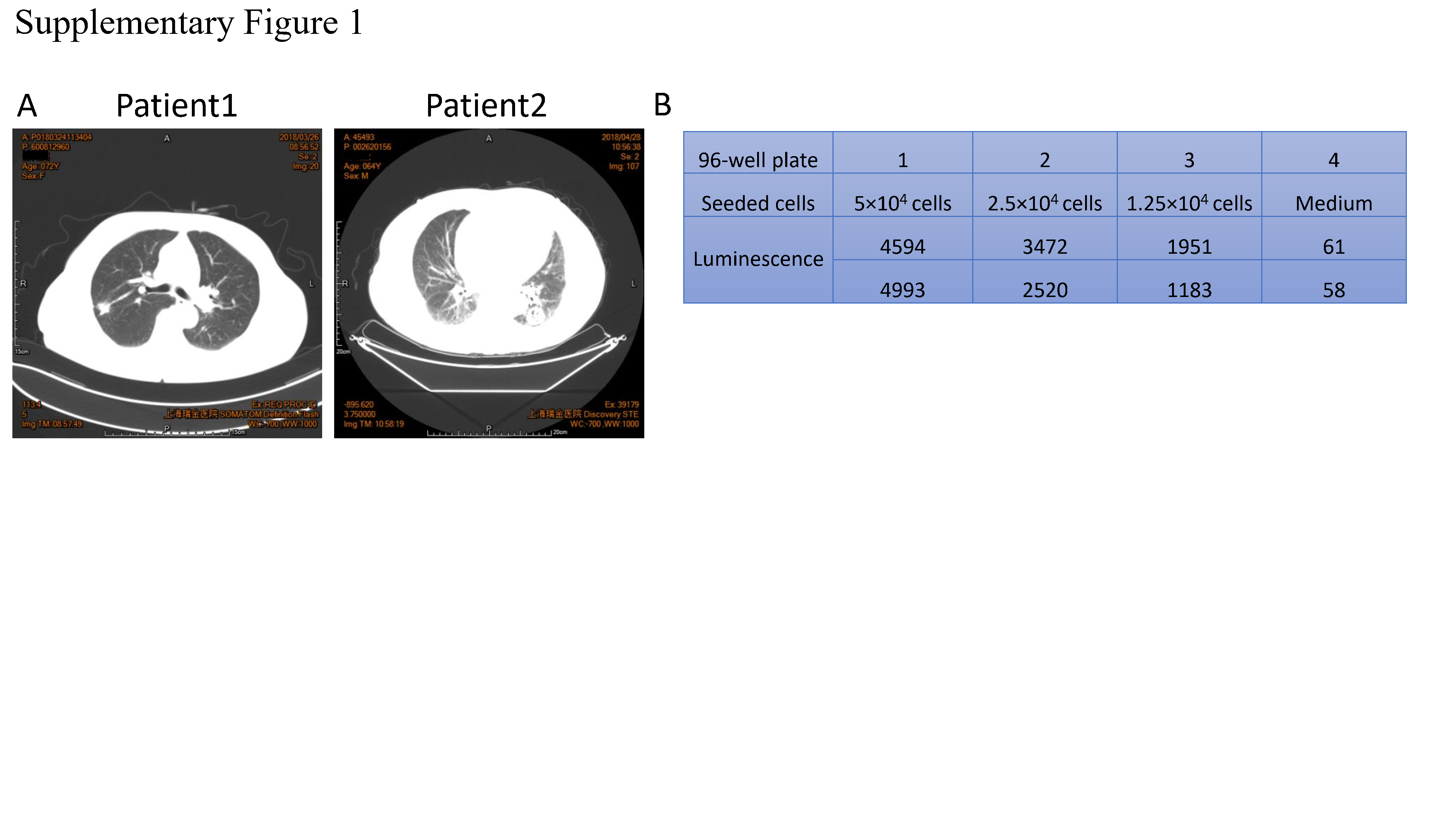

Supplement: Supplementary file 2 — Supplementary Fig.1 [file 41419_2020_2916_MOESM2_ESM.png]

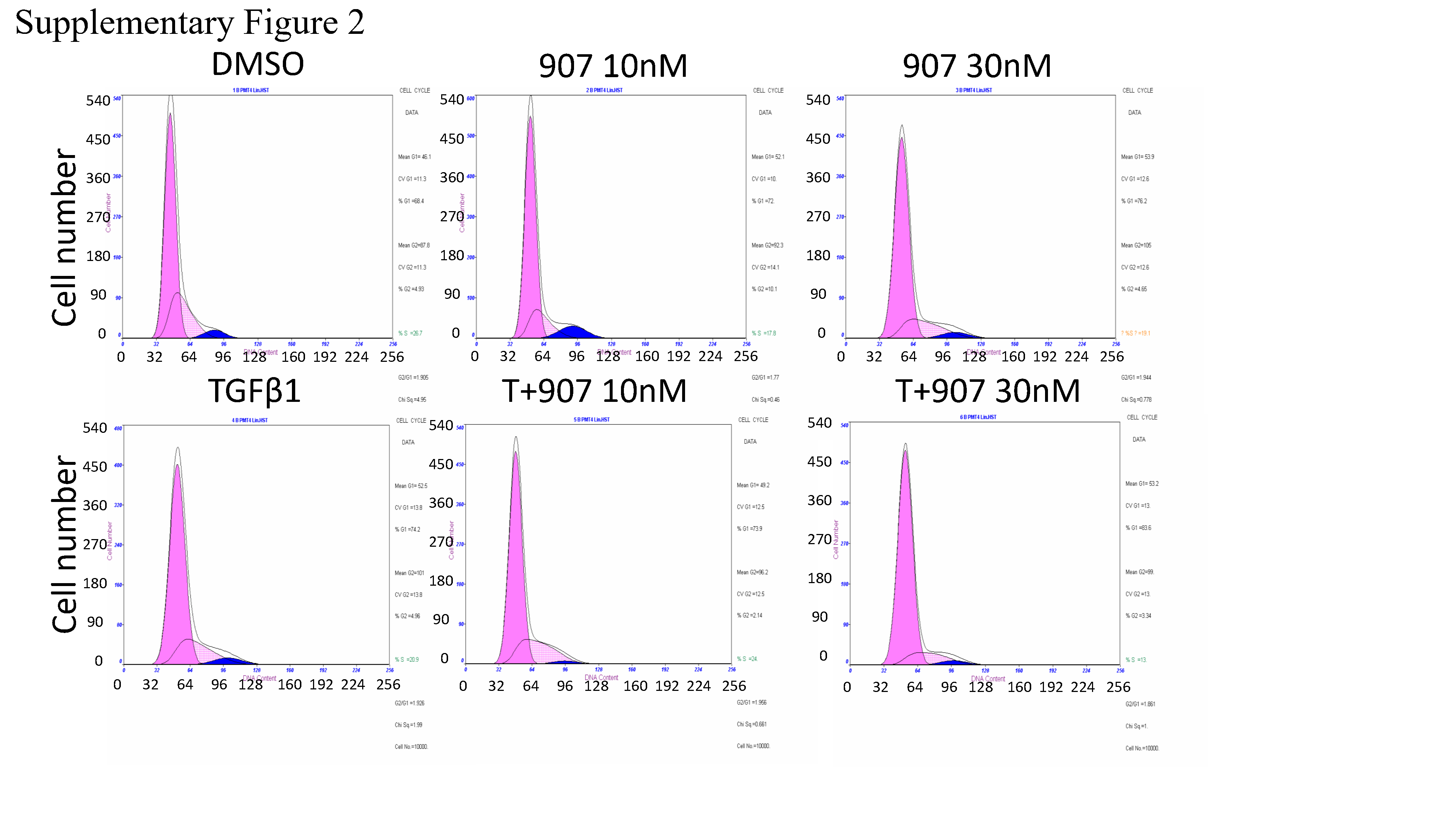

Supplement: Supplementary file 3 — Supplementary Fig.2 [file 41419_2020_2916_MOESM3_ESM.png]

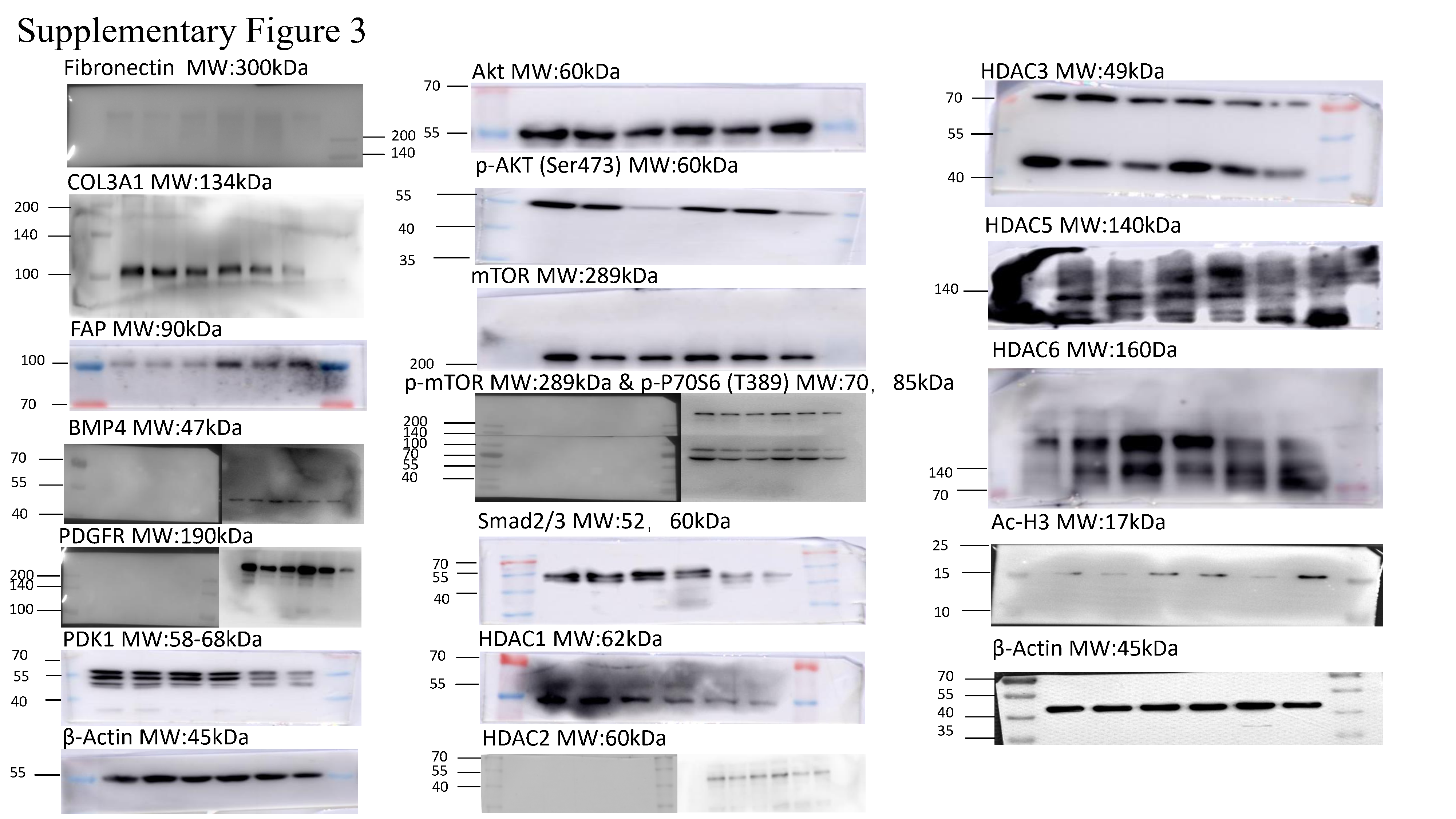

Supplement: Supplementary file 4 — Supplementary Fig.3 [file 41419_2020_2916_MOESM4_ESM.png]

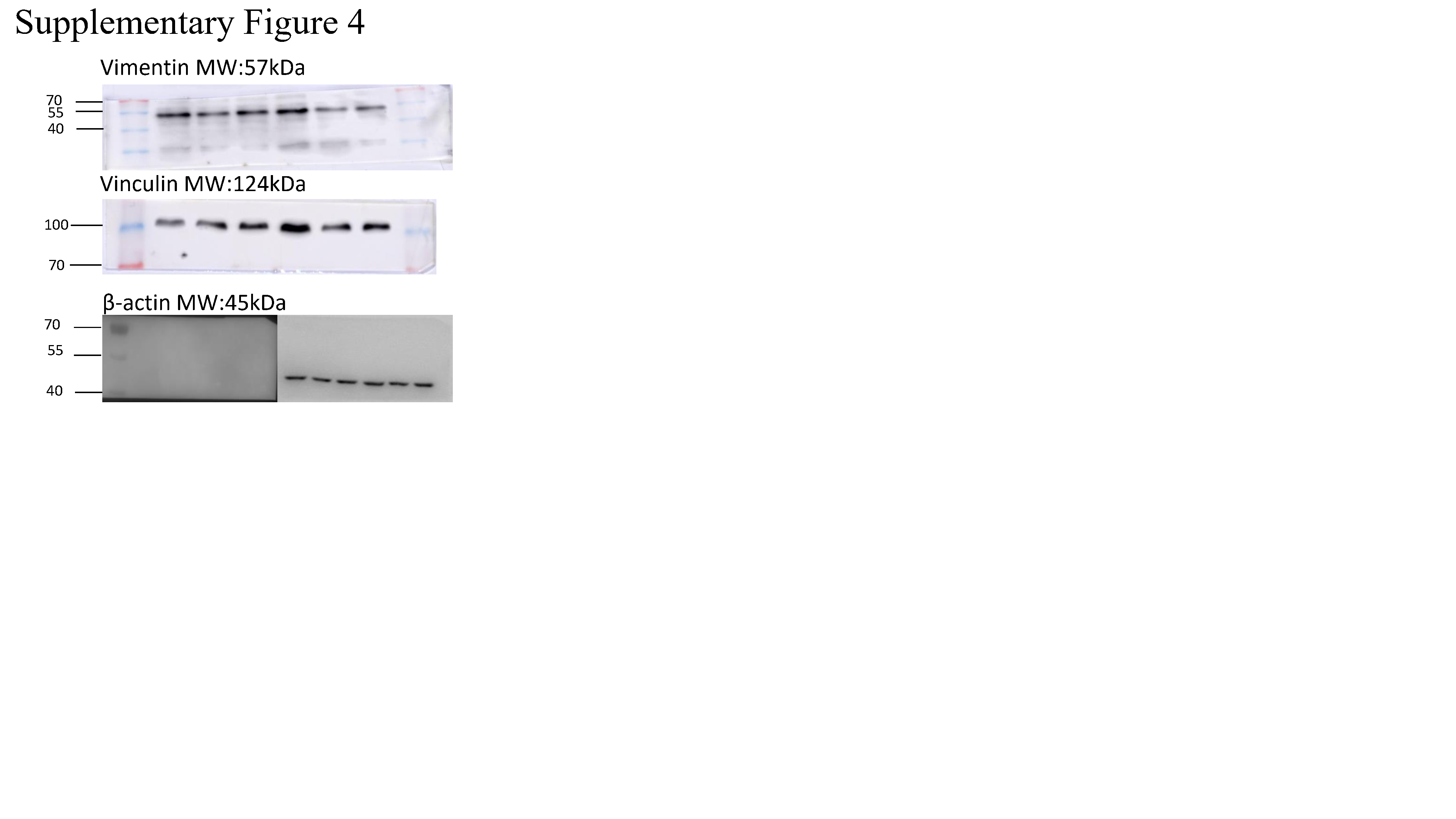

Supplement: Supplementary file 5 — Supplementary Fig.4 [file 41419_2020_2916_MOESM5_ESM.png]
